# Molecular Docking Study and 3D-QSAR Model for Trans-Stilbene Derivatives as Ligands of CYP1B1

**DOI:** 10.3390/ijms26031002

**Published:** 2025-01-24

**Authors:** Zbigniew Dutkiewicz, Renata Mikstacka

**Affiliations:** 1 Department of Chemical Technology of Drugs, Poznan University of Medical Sciences, Rokietnicka 3, 60-806 Poznań, Poland; 2 Department of Inorganic and Analytical Chemistry, Collegium Medicum, Nicolaus Copernicus University, Dr. A. Jurasza 2, 85-089 Bydgoszcz, Poland; mikstar@cm.umk.pl

**Keywords:** cytochrome P450, CYP1B1, molecular docking, 3D-QSAR, trans-stilbene derivatives

## Abstract

Scientific research on stilbenes is conducted for their chemopreventive and therapeutic properties. In experimental studies, natural and synthetic trans-stilbenes exhibit antioxidant, anti-inflammatory, cardioprotective, and anticancer effects. The antitumor activity of some natural and synthetic stilbenes is associated with their interaction with cytochrome P450 family 1, which leads to the inhibition of procarcinogen activation. In the present study, three-dimensional quantitative structure–activity relationship analysis (3D-QSAR) was performed on a series of forty-one trans-stilbene derivatives to identify the most significant features of the molecules responsible for their CYP1B1 inhibitory activity. The developed 3D-QSAR model presented a cross-validated correlation coefficient *Q*^2^ of 0.554. The model’s predictive ability was confirmed by external validation (*r*^2^ = 0.808). The information provided by 3D-QSAR analysis is expected to be valuable for the rational design of novel CYP1B1 inhibitors.

## 1. Introduction

Cytochromes P450 (CYPs) are a superfamily of hemoproteins exhibiting diverse monooxidase activities. Cytochrome 1B1 (CYP1B1) is a member of CYP family 1, consisting of three isozymes: CYP1A1, CYP1A2, and CYP1B1. These cytochromes attract interest as the enzymes participating in the procarcinogen activation and metabolism of numerous endogenous substances and xenobiotic compounds [1]. Their activity must be taken into account with regard to drug metabolism and potential drug–drug interactions. Moreover, CYP1B1, unlike other CYP1 isozymes, is involved in the carcinogenicity of 17-β-estradiol (E2) by metabolizing it to 4-hydroxyestradiol, which may form covalent bonds with DNA, leading to mutagenic effects [2]. In tumor tissues, the overexpression of CYP1B1 has been observed, and this has made the enzyme a promising target for anticancer prodrugs that require metabolic activation. On the other hand, CYP1B1 overexpression in cancer cells may be disadvantageous if this enzyme can metabolize the therapeutics to inactive forms.

Trans-stilbenes are a group of compounds containing *trans*-1,2-diphenylethene as the core of a molecule substituted with different organic groups, e.g., hydroxy or alkoxy groups, or conjugated with organic acids, polyphenols, or carbohydrates. These hydrophobic compounds penetrate the cell membrane and show antagonistic properties against the aryl hydrocarbon receptor (AhR) responsible for the upregulation of CYP1 isozymes [3,4].

Natural stilbenes are polyphenols, which are a widespread group of phytochemicals synthesized in plants as a result of damage, thermic shock, UV radiation, and pathogen attack. A number of 459 stilbenes from 45 plant families and from 196 plant species were identified [5]. The interest in bioactivities of stilbene derivatives was initiated by the discovery of the particular chemopreventive activity of trans-resveratrol (trans-3,4′,5-trihydroxystilbene)—a natural compound occurring in the human diet, present in berries, the skin of grapes, wine, and numerous medicinal plants [5]. So far, the effectiveness of resveratrol has not been proven in clinical trials [6]. However, experimental studies have shown the antioxidant, anti-inflammatory, cardioprotective, and antitumor activities of natural and synthetic trans-stilbenes [7,8]. The research on stilbenes concerning their chemopreventive and therapeutic properties was inspired by the need to find derivatives with potent effects characterized by better bioavailability, water solubility, and stability. Among natural analogs of resveratrol, pterostilbene (3,5-dimethoxy-4′-hydroxy-trans-stilbene) was widely investigated, exhibiting pharmacokinetic properties, as well as antioxidant and anticancer activities at all stages of carcinogenesis better than the parent compound [9,10]. In the ongoing search for pharmacologically active compounds with better pharmacokinetic parameters, a series of trans-stilbene derivatives are designed and synthesized.

The anticancer activity of some natural and synthesized stilbenes is involved in their interaction with cytochrome P450 family 1, leading to the inhibition of procarcinogen activation, which is responsible for the initiation phase of carcinogenesis [7]. The inhibitory activity of natural and synthetic stilbene derivatives towards CYP1B1 activity in relation to human health care has been demonstrated in numerous studies (see review [11]).

In light of this experimental basis, exploring the interactions of CYP1B1 ligands with the active site of the enzyme at the molecular level would be worthwhile. Computational methods are helpful, and for two decades they have been intensively developed to expand our knowledge of the SAR (structure and activity relationship) of cytochrome P450 inhibitors [12,13,14].

QSAR (quantitative structure–activity relationship) models are used in the natural sciences to predict the physicochemical properties of tested molecules. These methods are widely employed in drug design and the optimization of their pharmacokinetic properties [15]. QSAR models can be used in the virtual screening of ligand databases, and 3D-QSAR (three-dimensional quantitative structure–activity relationship) studies can be targeted to find CYP inhibitors or substrates with increased affinity to enzyme binding sites through knowledge-based design [12]. QSAR methods have also been used to search for selective ligands of CYP family isoenzymes: CYP1A1, CYP1A2, and CYP1B1 [13,16]. Natural compounds that inhibit CYP1 enzyme activity belong to flavonoids, naphtoflavonoids, steroids, benzochalcone derivatives, and stilbenoids [11]. CYP1 ligands include harmful compounds found in cigarette smoke and industrial pollution, such as polycyclic aromatic hydrocarbons, their diols, and heterocyclic aromatic compounds. The QSAR method and the CDOCKER [17] docking procedure were used to create CYP1A1 and CYP1B1 models to recognize the mechanism of procarcinogen activation [18]. Raju and coworkers identified CYP1B1 inhibitors with the use of the 3D-QSAR model and other computational methods [19]. The novel CYP1B1 inhibitors, α-naphthoflavone derivatives, were synthesized, and their structure–activity relationship was analyzed with 3D-QSAR methods [20]. Moreover, 2D-QSAR was employed to analyze the molecular descriptors that characterize the inhibitory activity of selected methoxy and methylthio-substituted trans-stilbene derivatives towards CYP1B1 [21]. Most recently, a 3D-QSAR model was employed to identify the most potent CYP1B1 inhibitors that were able to reverse docetaxel resistance in a CYP1B1-overexpressed MCF7 cell line [19,22]. The resistance of cancer cells to chemotherapy is a serious limitation in therapy effectiveness. Metabolism-associated drug inactivation is one of the resistance mechanisms to anticancer drugs [23]. Paclitaxel, docetaxel, tamoxifen, cisplatin, mitoxantrone, flutamide, and gemcitabine, the agents used in cancer chemotherapy, are metabolized by CYP1B1 [24,25,26,27], which is overexpressed in extrahepatic cancer tissues, including cancers of the breast, colon, lung, esophagus, skin, lymph node, brain, and testis [28]. This phenomenon significantly diminishes the amount of active drug in cancer tissues. Intensive research continues to be carried out to facilitate the design of new effective CYP1B1 inhibitors that would help to overcome CYP1B1-mediated resistance.

The present study aimed to develop a 3D-QSAR model applicable to the further search for *trans*-stilbene derivatives showing potent and selective CYP1B1 inhibition. Experimental data on the activity of trans-stilbenes as CYP1B1 inhibitors, available in the literature [29,30,31,32,33], provide an opportunity to use them to build predictive models for designing new efficient inhibitors from this group of compounds. The inhibitors of nanomolar activity already found among trans-stilbene derivatives [30,32] indicate the possibility of identifying further potent compounds. This paper presents a 3D-QSAR model based on a molecular docking study dedicated exclusively to trans-stilbene derivatives as CYP1B1 inhibitors.

## 2. Results and Discussion

### 2.1. Three-Dimensional Quantitative Structure–Activity Relationship Model

So far, no 3D-QSAR model has been created for trans-stilbene derivatives. A 2D-QSAR mode, based on twenty-four trans-stilbene derivatives as CYP1B1 inhibitors [21], was obtained using experimental data for trans-stilbene derivatives with methoxy or methylthio substituents as CYP1B1 inhibitors. This model showed the relationship between the inhibitory activities of tested compounds and the values of the descriptors Eig04_AEA (bo), MaxDD (2D descriptors), RDF070s, RDF035m, and Mor10m (3D descriptors). However, 3D-QSAR methods were used for other groups of compounds exhibiting inhibitory activity toward CYP1B1. A series of derivatives of α-naphthoflavone (ANF), a potent CYP1B inhibitor, were designed, synthesized, and assessed for their inhibitory properties by Dong et al. [16,20]. Molecular docking and 3D-QSAR helped to find structural features that may determine the inhibitory activity of studied compounds. Another 3D-QSAR model based on the data for compounds with diverse molecular skeletons, including flavonoids, trans-stilbenes, anthraquinones, coumarins, and alkaloids, was used by Raju et al. [19] for screening compounds with pyrrole-based chalcone scaffolds.

#### 2.1.1. Molecular Alignment

**Figure 1:**
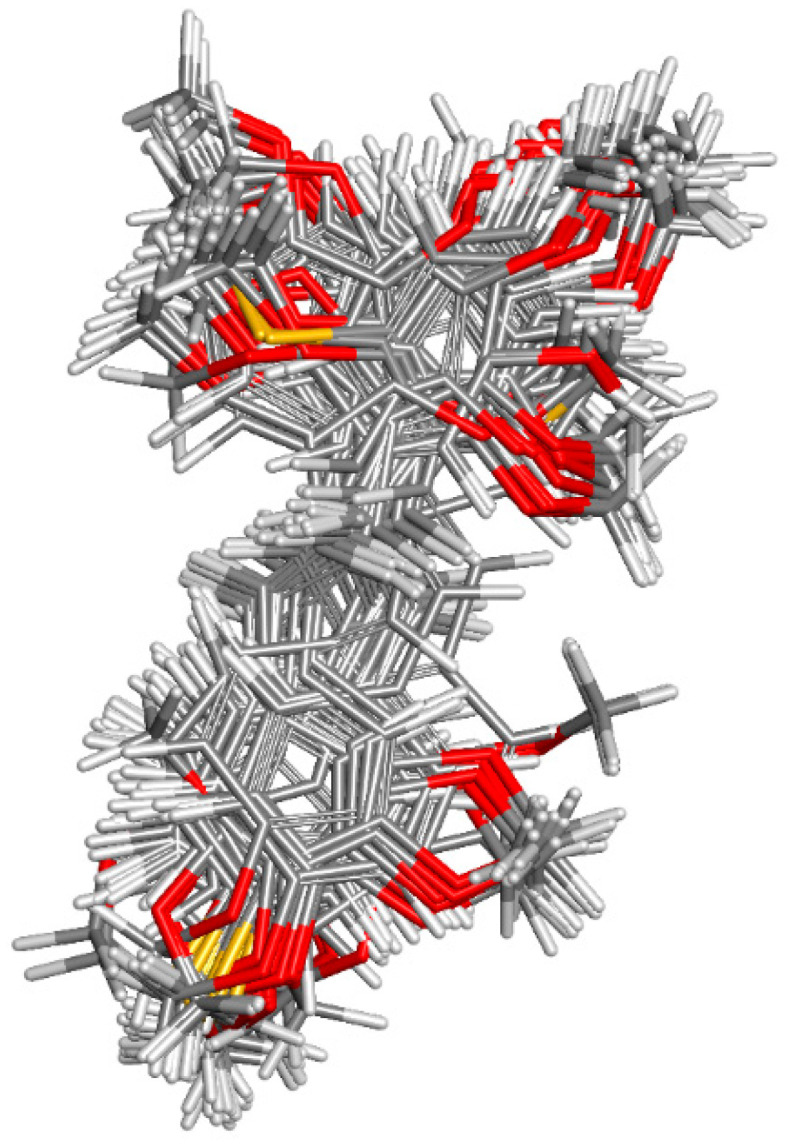
Alignment of the data set molecules for conformations generated by CDOCKER. Superimposed molecules are presented as stick models with gray carbon, red oxygen, yellow sulfur, and white hydrogen atoms.

Proper molecular alignment is crucial for successful 3D-QSAR modeling. In the study of Raju et al. [19], the alignment of compounds was obtained by the superposition of molecules using the pharmacophore model and the Maximum Common Substructure (MCS) method. In the study of Dong et al. [20], one of the most active molecules in the series was chosen as a template molecule, and all the remaining molecules were aligned with it using the database alignment method. The present study employed a structure-based design approach to find and align the possible binding conformations. For this purpose, all the training and test set molecules were docked into the CYP1B1 binding site with the CDOCKER procedure in Discovery Studio 2016. The alignment of molecules, obtained by superimposing the conformations with the highest scores, is shown in Figure 1 and was used to build the 3D-QSAR model.

#### 2.1.2. Model Validation

A three-dimensional quantitative structure–activity relationship study (3D-QSAR) of molecular field analysis using forty-one trans-stilbene derivatives was conducted to understand the relationship between structure and activity better. Table 1 contains the structural formulas of the compounds used in this work and their activity data in IC_50_ [29,30,31,32,33]. Of the 41 compounds, 33 were selected as the training set for model construction, and the remaining 8 were used as the test set (underlined in Table 1). The energy grids were computed using two probe types to measure electrostatic and steric effects. Experimental and predicted pIC_50_ values, together with the residual errors of all compounds for this QSAR model, are given in Table 1.

The internal validation of the model was performed using 5-fold cross-validation to obtain the optimum number of partial least squares (PLS) components. The highest value of the cross-validated correlation coefficient $Q^{2}$ = 0.554 was obtained for the three-component PLS model (Table 1). External validation using the test set showed that the obtained 3D-QSAR model meets the following conditions: $Q^{2}$ > 0.5, $r^{2}$ > 0.6, $r_{0}^{2}$ and $r_{0}^{\prime 2}$ close to the value of $r^{2}$, $\left(r^{2}-r_{0}^{2}\right)/r^{2}$ or $\left(r^{2}-r_{0}^{\prime 2}\right)/r^{2}$ < 0.1, 0.85 ≤ $k_{0}$ ≤ 1.15 or 0.85 ≤ $k_{0}^{\prime }$ ≤ 1.15, $\left|r_{0}^{2}-r_{0}^{\prime 2}\right|$ < 0.3, $r_{m\;(test)}^{2}$ > 0.5, and $r_{m\;(test)}^{\prime 2}$ > 0.5 [34,35,36,37,38]. This indicates the reliable predictive capability of the model. The results of internal and external validation are given in Table 2. For the external test set, $r_{0}^{2}$, $r_{0}^{\prime 2}$, $r_{m\;(test)}^{2}$, and $r_{m\;(test)}^{\prime 2}$ were 0.801, 0.795, 0.741, and 0.717, respectively, which indicates that the 3D QSAR model built in this study is acceptable, with good external predictability ($r_{m\;(test)}^{2}$ > 0.5) [37].

**Figure 2:**
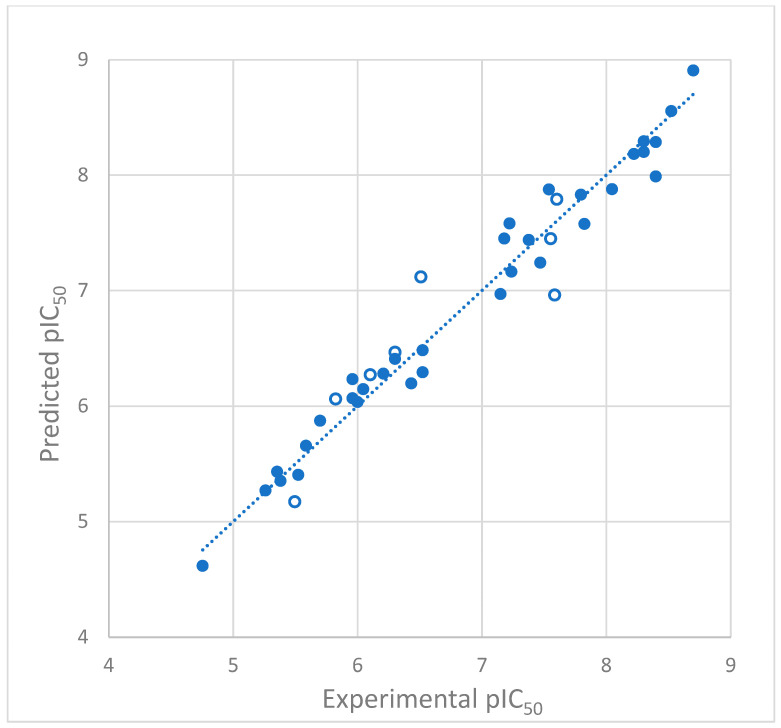
Plot of predicted vs. experimental values of pIC_50_ for the training set (filled circles) and test set (empty circles).

The correlation plot between the experimental and predicted inhibitory activity for the training set and the test set is shown in Figure 2.

In the developed 3D-QSAR model, good agreement was obtained between the experimental and predicted pIC_50_ values, which, with two exceptions, did not differ by more than 0.4 logarithmic units. The exceptions are compounds **12** and **23** from the test set, for which the error is about 0.6 log units (Table 1). However, none of the inhibitors were detected as outliers. The discrepancies between the experimental and predicted activity of the compounds (Figure 2) may depend on factors other than the enzyme–ligand affinity, such as ligand solubility in the enzyme environment, molecule hydration, or the different accessibility of entry channels. Higher than expected differences between experimentally determined and predicted activity may also result from the flexibility of cytochrome CYP1B1. The 3D-QSAR model was built based on the ligand poses obtained in docking to a rigid receptor. At the same time, cytochromes are flexible proteins that adapt their active site to binding molecules [39,40,41,42]. Such binding site flexibility is also demonstrated by cytochrome CYP1B1 [43]. The good correlation of experimental and predicted pIC_50_ values (Figure 2) and the fulfillment of the conditions required for reliable models with good predictive ability (Table 2) suggest that the obtained model is of sufficient quality and can be used to design new inhibitors with improved potency.

**Figure 3:**
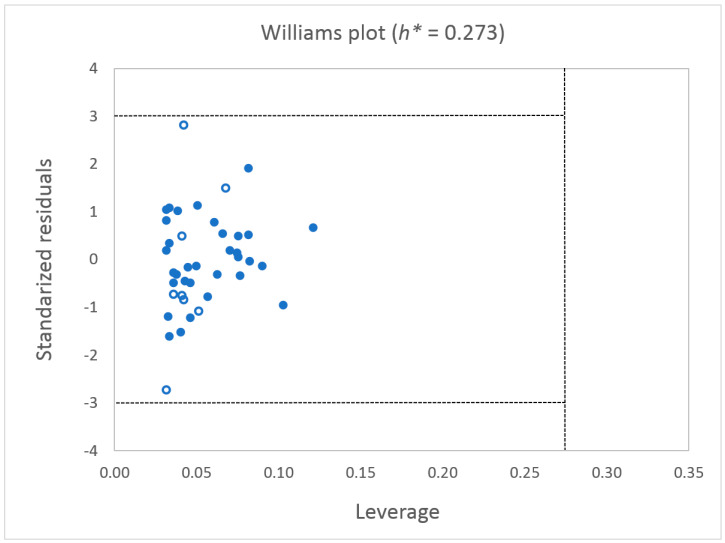
Williams plot for applicability domain of the 3D-QSAR model (training set—filled circles; test set—empty circles).

The leverage approach [44,45] was employed to determine the applicability domain (AD) of the developed 3D-QSAR model. In this case, AD presents a Williams plot, which shows the standardized residuals (σ) versus the leverage (*h*) (Figure 3). The boundaries for the applicability domain represent the horizontal dashed lines (±3 of standardized residuals) and one vertical dashed line corresponding to the value of the warning leverage h* = 0.273. As can be seen, two compounds, **12** and **23,** show relatively large standardized residuals, although still below the value of ±3σ. However, since none of the compounds in the test set exceed the cutoff value of leverage (h*), they all fall within the model’s AD.

#### 2.1.3. Contour Map Analysis

**Figure 4:**
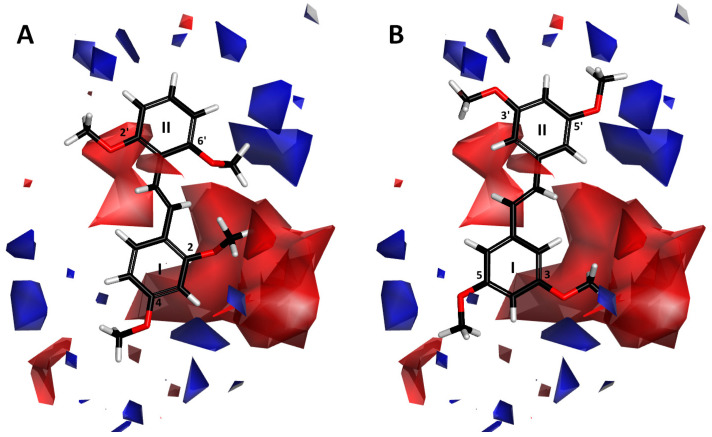
Electrostatic contour maps for 3Q-QSAR model with (**A**) compound **1** and (**B**) compound **41** aligned. Ligands are represented as stick models with black carbon, red oxygen, and white hydrogen atoms. Red and blue contours represent regions that favor high-electron-density and low-electron-density groups, respectively.

**Figure 5:**
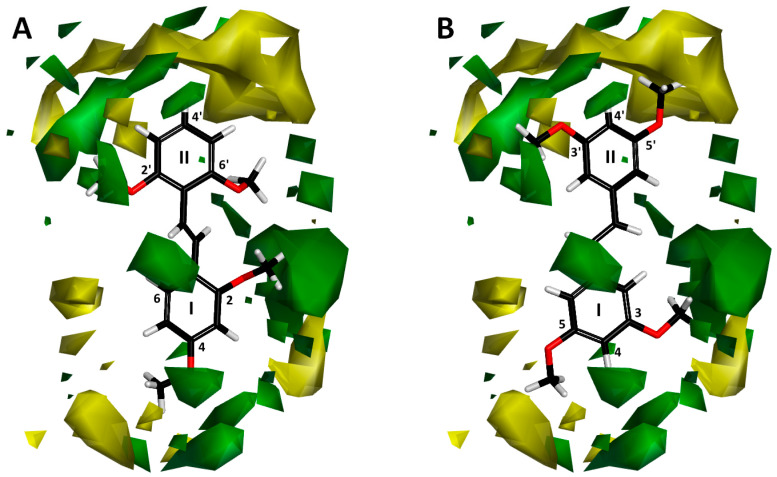
Steric contour maps for 3D-QSAR model with (**A**) compound **1** and (**B**) compound **41** aligned. Ligands are represented as stick models with black carbon, red oxygen, and white hydrogen atoms. Green and yellow contours represent regions where the presence of bulky groups may increase and decrease activity, respectively.

Two ligands that differ in activity, compounds **1** (IC_50_ = 2 nM) and **41** (IC_50_ = 17,600 nM), aligned with the electrostatic and van der Waals isosurfaces, are displayed in Figure 4 and Figure 5, respectively. The 3D-QSAR electrostatic contour map (Figure 4) shows red contours surrounding the trans-stilbene derivative molecules, representing areas where the ligand molecule’s high electron density (negative charge) may enhance activity. Blue-colored contours represent regions where increased electron deficiency (positive charge) favors inhibitory activity.

The large red contour near ring I indicates that atoms or groups with high electron density present here may play a favorable role in inhibitory potency. The proximity of the oxygen atom of the methoxy substituent at position 2 of compound **1** to this area may contribute to the increased activity of this compound (Figure 4A). The methyl group of this substituent is tilted out of the plane of ring I, which causes the exposure of the lone pairs of the oxygen atom to the red region. In the case of compound **41** (Figure 4B), the activity may be reduced by the unfavorable proximity of this red contour and the positively polarized methyl group of the substituent at position 3 (right side of ring I). A small red region is also observed near ring I, on the opposite side of the red area already described. Figure 4B shows that in its vicinity, there is a positively polarized methyl group of the substituent at position 5 of compound **41** (left side of ring I), which may impact the activity reduction in **41**. The next red area that may affect the activity of the tested inhibitors lies near ring II. Thus, in active compound **1** (Figure 4A)**,** the red region is adjacent to the electron-rich oxygen atom of the substituent at position 2′ (left side of ring II).

Blue contours are located around both rings, but they are much closer to ring II, and the largest of these areas is located on its right side (Figure 4). In active compound **1**, the methoxy substituent’s positively polarized methyl group at position 6′ (right side of ring II) is directed towards the blue region, which favors higher activity. In turn, in the low-active ligand **41**, the blue areas are adjacent to the oxygen atoms of methoxy substituents at positions 3′ and 5′ of ring II (Figure 4B), which is not favorable from the point of view of inhibitory activity towards CYP1B1.

The steric field map shows the regions where bulky substituents in the ligand molecule increase (green contours) or decrease (yellow contours) the activity. The contour map of the 3D-QSAR model (Figure 5) shows large green regions near the substituents at positions 2 (right side of ring I), 2′, and 3′ (left side of ring II). Small green contours are also visible near positions 4 and 6 of ring I and 4′ and 6′ of ring II. The disfavored steric regions are depicted in yellow (Figure 5). The extensive one is seen above ring II. The next small yellow contours are close to positions 3 and 5 of ring I.

In the case of active compound **1** (Figure 5A), the methyl groups of three substituents are directed towards the green areas; this is primarily the substituent in position 2 (ring I) and the groups in positions 2′ and 6′ (ring II). This orientation of the CH_3_ groups is favorable for activity. In compound **41** (Figure 5B), methyl groups of only two substituents are located near the green areas, in positions 3 (on the right side of ring I) and 3′ (on the left side of ring II). In turn, the methyl group at position 5′ (right side of ring II) is very close to the yellow region, contributing to the activity reduction. It is worth noting that the methyl groups of the substituents in position 4 (ring I) of compound **1** and position 5 (ring I) of **41** are directed towards the same yellow region (Figure 5). Nevertheless, the methyl group in the less active compound is much closer to this region (Figure 5B), which may cause a decrease in its activity.

**Figure 6:**
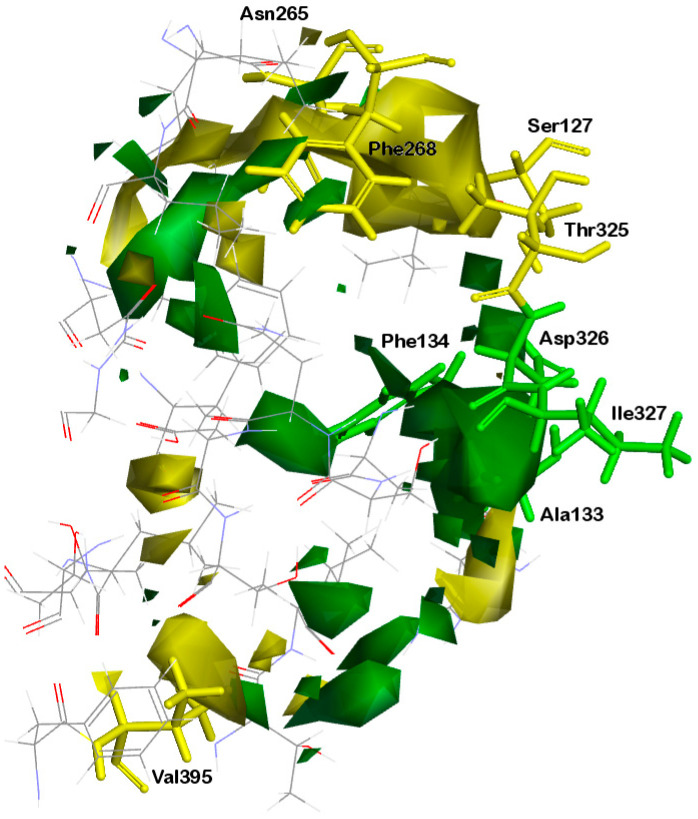
Steric field of 3D-QSAR model and amino acids constituting CYP1B1 binding site. Yellow areas responsible for unfavorable steric interactions are surrounded by amino acids depicted as yellow sticks; those represented as green sticks are adjacent to the green contour, where groups with steric bulk enhance activity. The remaining amino acids forming the binding site are shown as lines with gray carbon, red oxygen, blue nitrogen and white hydrogen atoms.

The presence of yellow areas, a large one located above ring II of ligands **1** and **41** and a smaller one near the substituent at position 5 of compound **41** (Figure 5), can be explained by analyzing the shape of the binding site. The mentioned yellow areas are due to the limitation of the space available to the ligands by the amino acids Ser127, Asn265, Phe268, and Thr325, as well as the presence of the branched side chain of Val395 (Figure 6). On the other hand, the green area around substituent 2 in compound **1** (ring I) is a pocket formed by amino acids Ala133, Phe134, Asp326, and Ala330 (Figure 6).

### 2.2. Docking Analysis

In the present study, the tested ligands—41 derivatives of trans-stilbene with methoxy and methylthio substituents (Table 1)—were docked into the active site of cytochrome CYP1B1 to predict their binding modes, analyze interactions with the enzyme, and to obtain the proper alignment of molecules for 3D-QSAR model construction As previously mentioned, 3D-QSAR studies on CYP1B1 so far have not only focused on other molecular skeletons than trans-stilbene, i.e., flavonoids and naphthoflavonoids [16,20], or compounds with a pyrrole-based chalcone scaffold [19,22], but also used a different method of molecule fitting: not docking, but superimposing other molecules onto the pharmacophore model or the selected structure of a highly active compound.

#### 2.2.1. Orientation of Ligands in the Binding Site

A co-crystallized α-naphthoflavone (ANF) ligand was also docked to the CYP1B1 binding site according to the described procedure to verify docking accuracy. A comparison of the docked ligand poses with the conformation observed in the crystal showed that the lowest RMSD value between the docked ANF pose and the experimental conformation, equal to 0.53 Å for heavy atoms, was obtained for the pose with the highest CDOCKER energy value. The low RMSD value confirms the reliability of the CDOCKER procedure in predicting the correct ligand pose for CYP1B1.

Among the docked derivatives, several substitution patterns exist in a given ring. These are, for example, rings with one substituent present in positions 2, 3, or 4; disubstituted rings in positions 2 and 4, 3 and 4, or 3 and 5; and compounds with an unsubstituted ring (Table 1). For the analysis of the orientation and interactions of ligands in the CYP1B1 active site, the poses with the highest CDOCKER energy value were selected. When analyzing the poses of ligands, special attention was paid to which ring was directed towards the heme (Supplementary Materials; Table S1). This allowed us to notice certain relationships between the substitution pattern in the ring and its distance from the heme.

**Figure 7:**
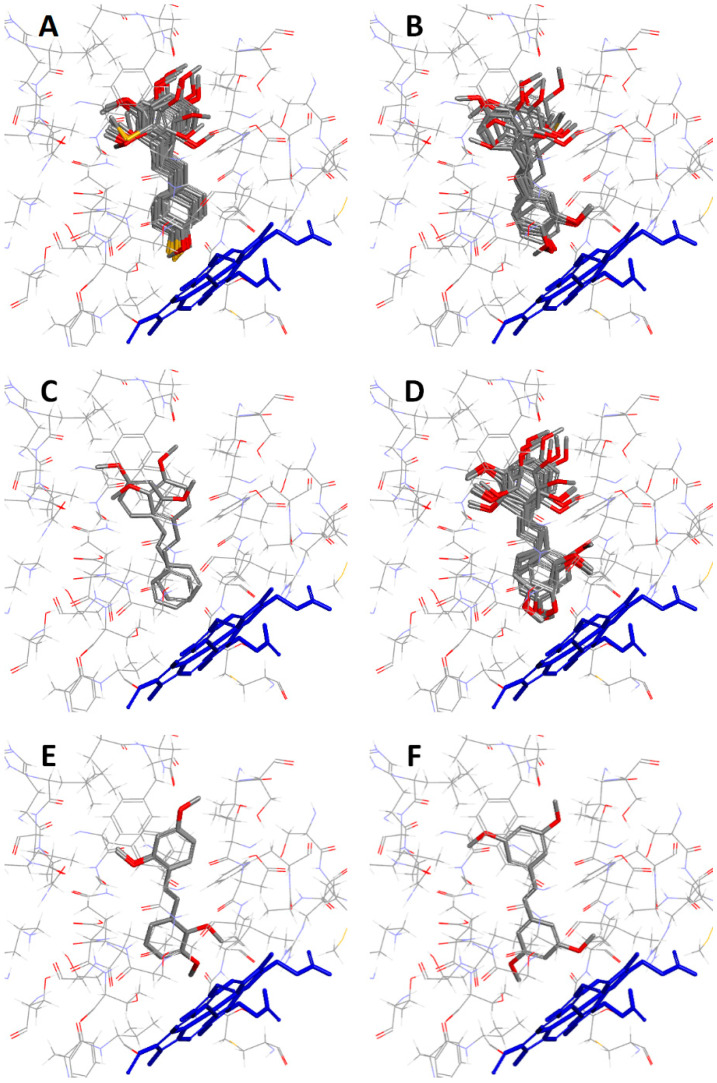
Ligands directed towards heme with (**A**) 4-monosubstituted, (**B**) 3,4-disubstituted, (**C**) unsubstituted, (**D**) 2,4-disubstituted, (**E**) 2,3-disubstituted or 3-monosubstituted, and (**F**) 3,5-disubstituted ring. Heme is shown as a stick model in blue.

As derivatives with a monosubstituted ring, with a methoxy or methylthio substituent in position 4 or 4′, compounds **12**, **13**, **15**, **17**, **19**, **25**, **26**, **28**, **29**, **30**, **31**, **32**, **33**, **34**, **35,** and **40** (Table 1) always orient themselves with this ring towards the heme (Figure 7A).

The compounds **12**, **13**, **15**, **17**, **19**, **25**, **26**, **28**, **29**, **30**, **31**, **32**, **33**, **34**, **35**, and **40** (Table 1), as derivatives containing a monosubstituted ring (with a methoxy or methylthio substituent in position 4 or 4′), always orient themselves with this ring towards the heme (Figure 7A).

The next group consists of compounds with a 3,4- or 3′,4′-dimethoxy-substituted ring, i.e., compounds **3**, **14**, **16**, **21**, **22**, **23**, **24**, **27**, **36**, **38,** and **39** (Table 1). Almost all these molecules point their 3,4-disubstituted ring towards the heme (Figure 7B). The exception is compound **21** (Table 1), which faces the heme with its unsubstituted ring (Figure 7C).

The orientation of molecules with a ring substituted in positions 2 and 4 or 2′ and 4′ with two methoxy groups depends on the substitution pattern in the second ring. Of the 15 molecules of such compounds, only 9 direct their 2,4-disubstituted ring towards the heme (Figure 7D). The rest turn towards the heme with their second ring. So in compounds **17** and **34** (Table 1), it is a ring substituted in position 4′ (Figure 7A); in **16** (Table 1), a ring substituted in positions 3 and 4 (Figure 7B); in **11** (Table 1), an unsubstituted ring (Figure 7C); and in the case of **9** and **18** (Table 1), a ring containing substituents in position 3′ or 2′, 3′ (Figure 7E).

Of the six compounds with a 3,5-dimethoxy-substituted ring (Table 1), only one ligand, compound **41**, has this ring facing the heme (Figure 7F). The remaining ligands (**7**, **28**, **33**, **36**, **37**) turn towards the heme with their second ring (Supplementary Materials; Table S1).

All the analyzed derivatives with an unsubstituted ring or a ring substituted only in the 4-position prefer the orientation in which this ring is directed towards the heme. Also, derivatives with a 3,4-dimethoxy substituted ring orient it towards the heme, the only exception being derivative **21** (Table 1), in which there is also an unsubstituted ring in the molecule.

The described preferences in the orientation of the rings determine the position of the other derivatives containing the mentioned molecular fragments. Therefore, the orientation of the ligands with dimethoxy-substituted rings in positions 2 and 4 or 3 and 5 depends on whether their molecules also contain unsubstituted, 4-monosubstituted, or 3,4-disubstituted rings.

#### 2.2.2. Protein–Ligand Interactions

**Figure 8:**
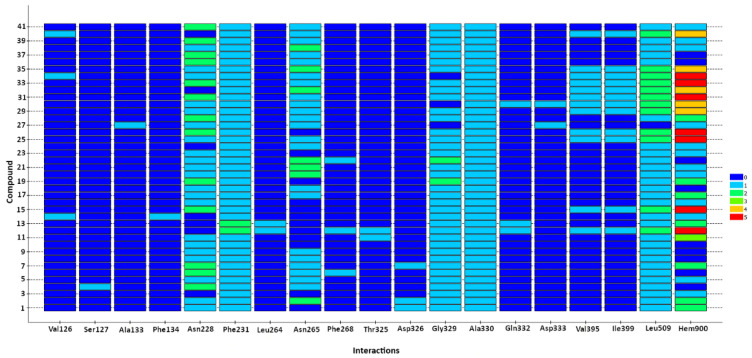
Heat map of CYP1B1–ligand interactions, representing number of compound interactions with a given residue: amino acid or heme (Hem900). The number of interactions with residue is color-coded: the minimum value (no interaction) is blue, the maximum (five interactions) is marked in red, and values from 1 to 4 are represented by colors from cyan to orange.

The amino acids that most frequently interact with ligands (Figure 8) are Phe231 (pi–pi stacking with all ligands), Ala330 (pi–alkyl interaction with all ligands), Gly329 and Ala330, with their amide/peptide bonds made via amide–pi stacking interactions (37 of 41 ligands), and Leu509 (mainly pi–alkyl interactions (40 of 41 ligands), but also alkyl–alkyl interactions with monosubstituted derivatives with a SCH_3_ group in position 4 (12 of 41 ligands)). Additionally, Asn228 (34 of 41 ligands) and Asn265 (29 of 41 ligands) are acceptors of weak hydrogen bonds (C-H·O) between ligands and proteins.

**Figure 9:**
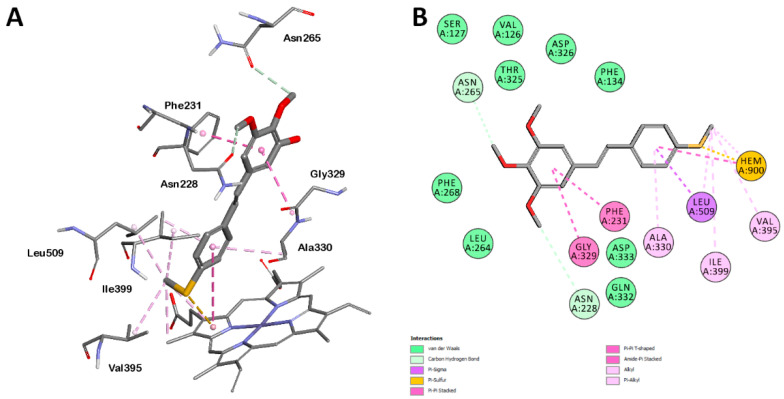
Interactions of ligand **35** with amino acids and heme: (**A**) 3D representation; (**B**) 2D diagram.

The heme also participates in numerous hydrophobic interactions (Figure 8). It is involved in pi–pi (T-shaped), pi–sulfur, pi–alkyl, and also alkyl–alkyl interactions between the methyl group of heme and the methylthio substituent at position 4 of the ring of the analyzed ligands. The example of ligand **35** shows the interactions of 4-methylthio derivatives with CYP1B1 (Figure 9).

The analysis of interactions between the ligands and the CYP1B1 binding site does not clearly indicate that the presence or absence of some interactions determines the inhibitory activity of the ligand. However, for some groups of ligands, it is possible to identify certain interactions that are characteristic of them. Thus, derivatives with a methylthio group at position 4 of the ring are characterized by simultaneous interaction with Val395, Ile399, and Leu509 (Figure 8). Molecules of these compounds also often interact with the heme, having four to five interactions with this cofactor. In comparison, other ligands do not interact with the heme or have only 2–3 interactions (Figure 8). Some strong inhibitors, such as compounds **1**, **2,** and **7** (Table 1), interact with Asp326 as an acceptor of weak hydrogen bonds (Figure 8).

In summary, forty-one trans-stilbene derivatives were studied in this work, using methods of searching for quantitative structure–activity relationships other than those previously used for this group of compounds, taking into account molecular fields and ligand conformations obtained in docking to CYP1B1. By docking ligands to the CYP1B1 active site to obtain their proper alignment, our 3D-QSAR model explains the activity of trans-stilbene derivatives by referring not only to the structure of their molecules, but also to the features of the binding site. The information provided by the 3D-QSAR model is encoded in the values of the steric and electrostatic fields surrounding the ligand molecules. Our model indicates the importance of the complementarity of the ligand shape and the binding site and may be used in further research for potent and selective CYP1B1 inhibitors.

## 3. Materials and Methods

### 3.1. Data Set

Forty-one CYP1B1 cytochrome inhibitors—trans-stilbene derivatives with methoxy (OMe) or methylthio (SMe) substituents—were used to build the 3D-QSAR model. Data on the inhibitory activity and the chemical structures of the 41 CYP1B1 inhibitors (Table 1) were collected from previous reports [29,30,31,32,33].

### 3.2. Molecular Docking

Three-dimensional structures of ligands were built in Discovery Studio 2016 (DS2016) [46] and subjected to energy minimization under a CHARMm force field with a root -mean-squared (RMS) difference of energy gradient reaching 0.1 kcal/mol Å.

Then, trans-stilbene derivatives were docked to the active site of CYP1B1 (PDB ID: 3pm0) using the CDOCKER procedure. The automatic Prepare protein procedure prepared the receptor for docking by removing water molecules, adding hydrogen atoms, and protonating amino acid residues at the specified pH (pH = 7.4). The binding site was defined as a sphere with a radius of 10 Å, based on the position of the protein-bound α-naphthoflavone (ANF). After defining the binding site, the molecule of ANF was removed from the structure. Calculations during docking were performed using the CHARMm force field, and partial charges for protein atoms and ligands were assigned according to the Momany–Rone (MR) method. First, using molecular dynamics, twenty conformations were generated for each ligand, each placed in the active site in ten different orientations by random rotations and translations. Subsequently, poses obtained this way were used for docking and further simulated annealing, minimization, and scoring. For the molecular dynamics analysis and simulated annealing, default values of parameters were used. Finally, poses were minimized with the complete CHARMm forcefield expression, and ten poses with the highest score were saved for each ligand after docking.

### 3.3. 3D-QSAR Modeling

The validation of the docking procedure for ANF showed that the docked pose most similar to the ligand position in the X-ray structure (PDB ID: 3pm0) is the one with the highest value of the scoring function of CDOCKER energy. Therefore, the same rule was used to select the active conformation for each tested ligand. The superimposed conformations of 41 trans-stilbene derivatives were then used to build and assess the quality of a 3D-QSAR model.

Compounds were split into training and test sets (underlined in Table 1) using the Diverse molecules method in DS2016. The molecular solvent accessible surface area (SASA) parameter was added to the default predefined set of molecular parameters to select a diverse subset of ligands. The SASA of the ligand was identified as one of the descriptors correlating with the activity of ligands towards CYP1B1 [17]. The training set percentage was set to 80, which ensured that 80% of the compounds were used to build the model (33 compounds), and the rest were used for external validation (8 compounds). The pIC_50_ scale (−logIC_50_), in which a higher value indicates greater potency, was used to measure the inhibitory activity.

For aligned ligands of the training set placed in a three-dimensional grid space, the Create 3D QSAR Model procedure available in Discovery Studio 2016 calculated the values of the electrostatic and steric fields on every grid point and built the partial least squares 3D-QSAR model using energy grids as descriptors. Electrostatic interactions were tested using a test charge of +1 (proton charge) and steric interactions (van der Waals) using a carbon atom (1.73 Å radius) as a probe. The test charge and the carbon atom were placed at grid points spaced by a constant distance of 1.5 Å (along each XYZ axis). Partial charges defined according to the rules of the Charmm force field were used for the ligands. The quality of the 3D-QSAR model was evaluated by internal 5-fold cross-validation and an external test set.

## 4. Conclusions

A molecular docking study of forty-one trans-stilbene derivatives was performed at the binding site of CYP1B1, allowing the prediction of their binding modes. Ligand alignment based on the docking study resulted in a satisfactory 3D-QSAR model with a cross-validated correlation coefficient (*Q*^2^ = 0.554) and good external predictability ($r^{2}=0.808,\;r_{m\;(test)}^{2}$ = 0.741).

Based on the docking results, it was found that the ligand’s orientation in the CYP1B1 binding site correlates quite well with the substitution patterns in both rings. The presence of rings substituted in a particular way often determines the orientation of the ligand, and some of these patterns have a dominant influence on the orientation of the ligand that is adopted in the binding site. Thus, depending on the number of substituents and their location, ligands are directed towards the heme, with the ring (i) substituted with only one group in position 4, (ii) 3,4-disubstituted, or (iii) unsubstituted. The orientation of other derivatives, including 2,4-disubstituted, depends on whether the second ring meets conditions (i–iii).

The 3D-QSAR contour maps provided important information to understand the structure–activity relationship and determine the structural conditions resulting in the increased inhibitory activity of the compounds. From the 3D-QSAR contours, we can see the following: (1) The surrounding of ligands with yellow and green contours indicates that the matching of the molecular shape and binding site is important for the activity of the tested derivatives, allowing the ligands to avoid the yellow areas and to place the substituents closer to the green contours. (2) A green contour near the substituents at C-2 and C-4 of ring I may indicate that introducing bulky groups at these positions may be helpful to enhance biological activity. The proximity of the green contour to the substituent at position C-2′ or C-3′ (ring II) is also beneficial for activity. (3) Yellow contours, one located above the C-5′ position (ring II) and another near the C-5 position (ring I), indicate that compounds with bulky groups at these regions may show reduced ability to inhibit CYP1B1 activity. (4) The electronegative group at C-2 (ring I) is favorable and may enhance inhibition potency.

The results of the docking and the analysis of 3D-QSAR help to comprehend the SAR of trans-stilbene derivatives and provide useful information for the rational design of novel derivatives as CYP1B1 inhibitors.

## Figures and Tables

**Table 1:** Experimental (exp) and predicted (pred) activities (pIC_50_) in mol/dm^3^, with residual values for training and test set (underlined) compounds (Cmpd).

Cmpd	Structure	IC_50_ (nM)	pIC_50_ (exp)	pIC_50_ (pred)	Residual
**1**	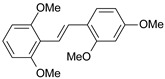	2	8.699	8.906	−0.207
**2**	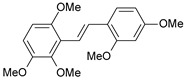	3	8.523	8.555	−0.032
**3**	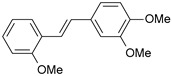	4	8.398	8.287	0.111
**4**	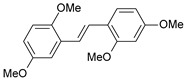	4	8.398	7.989	0.409
**5**	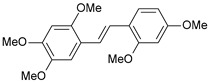	5	8.301	8.201	0.100
**6**	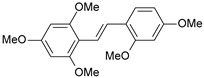	5	8.301	8.293	0.008
**7**	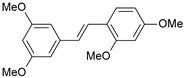	6	8.222	8.183	0.039
**8**	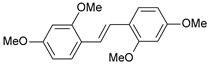	9	8.046	7.879	0.167
**9**	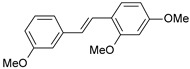	15	7.824	7.578	0.246
**10**	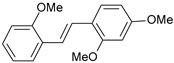	16	7.796	7.831	−0.035
**11**	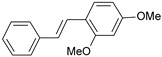	25	7.602	7.790	−0.188
**12**	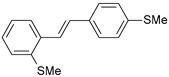	26	7.585	6.962	0.623
**13**	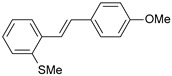	28	7.553	7.450	0.103
**14**	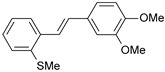	29	7.538	7.876	−0.338
**15**	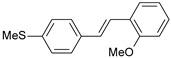	34	7.469	7.243	0.226
**16**	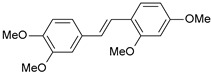	42	7.377	7.440	−0.063
**17**	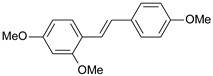	58	7.237	7.165	0.073
**18**	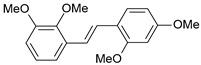	60	7.222	7.582	−0.360
**19**	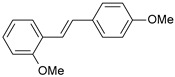	66	7.180	7.451	−0.271
**20**	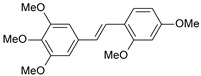	71	7.149	6.971	0.178
**21**	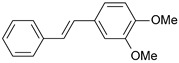	300	6.523	6.484	0.039
**22**	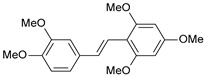	300	6.523	6.294	0.229
**23**	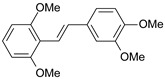	310	6.509	7.119	−0.610
**24**	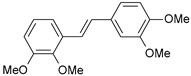	370	6.432	6.197	0.235
**25**	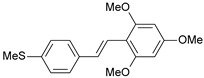	500	6.301	6.467	−0.166
**26**	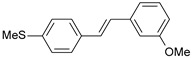	500	6.301	6.409	−0.108
**27**	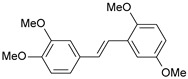	620	6.208	6.281	−0.073
**28**	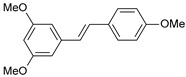	790	6.102	6.272	−0.169
**29**	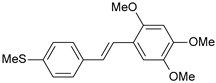	900	6.046	6.147	−0.101
**30**	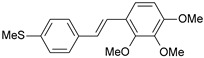	1000	6.000	6.036	−0.036
**31**	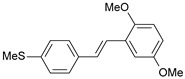	1100	5.959	6.234	−0.275
**32**	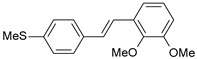	1100	5.959	6.068	−0.109
**33**	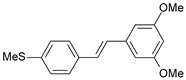	1500	5.824	6.062	−0.238
**34**	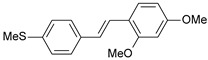	2000	5.699	5.873	−0.174
**35**	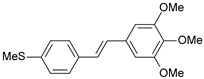	2600	5.585	5.657	−0.072
**36**	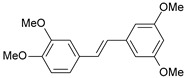	3000	5.523	5.405	0.118
**37**	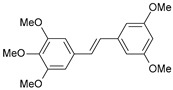	3200	5.495	5.172	0.323
**38**	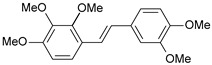	4170	5.380	5.353	0.027
**39**	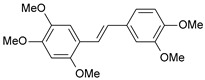	4440	5.353	5.432	−0.079
**40**	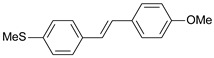	5500	5.260	5.270	−0.010
**41**	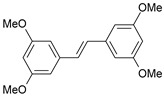	17,600	4.754	4.617	0.137

**Table 2:** Statistical parameters for the validation of the 3D-QSAR model ^(^*^)^.

Parameter	3D-QSAR Model	Threshold Value
$Q_{\left(5-fold\right)}^{2}$	0.554	>0.5
$r^{2}$	0.808	>0.6
$r_{0}^{2}$	0.801	$\mathrm{Close}\;\mathrm{to}\;\mathrm{the}\;\mathrm{value}\;\mathrm{of}\;r^{2}$
${r\prime }_{0}^{2}$	0.795	$\mathrm{Close}\;\mathrm{to}\;\mathrm{the}\;\mathrm{value}\;\mathrm{of}\;r^{2}$
$\left(r^{2}-r_{0}^{2}\right)/r^{2}$	0.009	<0.1
$\left(r^{2}-r_{0}^{\prime 2}\right)/r^{2}$	0.016	<0.1
$\left|r_{0}^{2}-r_{0}^{\prime 2}\right|$	0.007	<0.3
$k_{0}$	0.993	$0.85\;\leq \;k_{0}$ ≤ 1.15
$k_{0}^{\prime }$	1.005	$0.85\;\leq \;k_{0}^{\prime }$ ≤ 1.15
$r_{m\;(test)}^{2}$	0.741	>0.5
$r_{m\;(test)}^{\prime 2}$	0.717	>0.5

^(^*^)^ $Q_{\left(5-fold\right)}^{2}$ is the squared 5-fold cross-validated correlation coefficient for the training set. The remaining parameters refer to the test set: $r^{2}$ is a squared correlation coefficient between observed and predicted values with intercept, $r_{0}^{2}$ is a squared correlation coefficient between observed and predicted values without intercept, and $k_{0}$ is the slope of the corresponding fitted line. $r_{0}^{\prime 2}$ and $k_{0}^{\prime }$ have the same meaning as $r_{0}^{2}$ and $k_{0}$ but use reversed axes (plot of predicted against observed values) [34,35]; $r_{m\;(\mathrm{test})\;}^{2}=r^{2}\left(1-\sqrt{r^{2}-r_{0}^{2}}\;\right)$ describes the closeness between the $r^{2}$ and $r_{0}^{2}$ determination coefficients; and $r_{m\;(\mathrm{test})\;}^{\prime 2}=r^{2}\left(1-\sqrt{r^{2}-r_{0}^{\prime 2}}\;\right)$ describes the closeness between the $r^{2}$ and $r_{0}^{\prime 2}$ determination coefficients [36,37,38].

## Data Availability

Dataset available on request from the authors.

## Supplementary Materials

The following supporting information can be downloaded at: https://www.mdpi.com/article/10.3390/ijms26031002/s1.
