# Supplementary material for: Molecular Docking Study and 3D-QSAR Model for Trans-Stilbene Derivatives as Ligands of CYP1B1
Source: Int J Mol Sci. 2025 Jan 24;26(3):1002. doi: 10.3390/ijms26031002 (PMC11817148; doi:10.3390/ijms26031002)
Supplement: Supplementary file 1 [file ijms-26-01002-s001.zip › ijms-3414081-supplementary.pdf]

Supplementary Materials

Molecular docking study and 3D-QSAR model for trans-stilbene derivatives as ligands of CYP1B1

Zbigniew Dutkiewicz[1] and Renata Mikstacka[2]

[1] Department of Chemical Technology of Drugs, Poznan University of Medical Sciences, Rokietnicka 3, 60-806 Poznań, Poland; zdutkie@ump.edu.pl
[2] Department of Inorganic and Analytical Chemistry, Nicolaus Copernicus University, Collegium Medicum, Dr. A. Jurasza 2, 85-089 Bydgoszcz, Poland; mikstar@cm.umk.pl

* Correspondence: zdutkie@ump.edu.pl

Keywords: cytochrome P450, CYP1B1, 3D-QSAR, trans-stilbene derivatives.

Table S1. Ligand orientation relative to heme.

Compound	Structure	Ring close to HEME
**1**	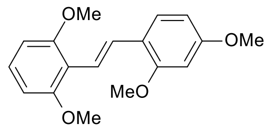	2,4-disubstituted
**2**	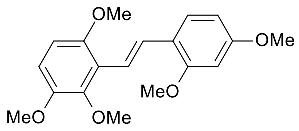	2,4-disubstituted
**3**	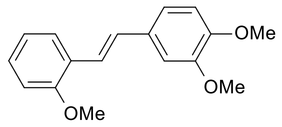	3,4-disubstituted
**4**	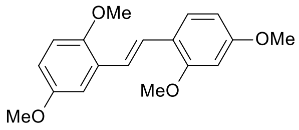	2,4-disubstituted
**5**	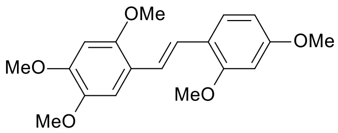	2,4-disubstituted

Compound	Structure	Ring close to HEME
**6**	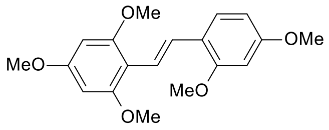	2,4-disubstituted
**7**	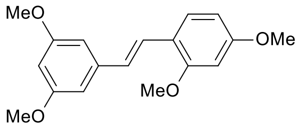	2,4-disubstituted
**8**	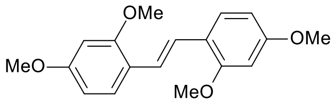	2,4-disubstituted
**9**	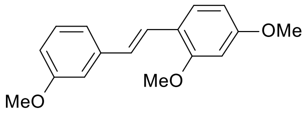	3′-monosubstituted
**10**	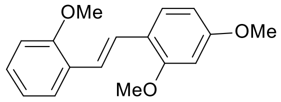	2,4-disubstituted
**11**	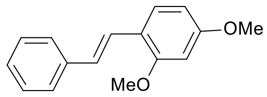	unsubstituted
**12**	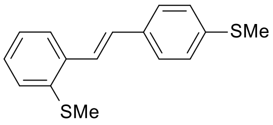	4′(SMe)-substituted
**13**	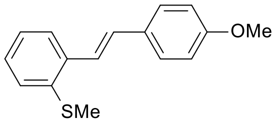	4′(OMe)-substituted
**14**	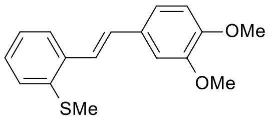	3,4-disubstituted
**15**	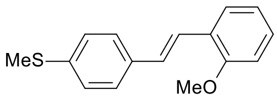	4′(SMe)-substituted
**16**	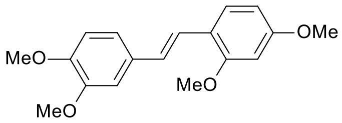	3,4-disubstituted

Compound	Structure	Ring close to HEME
**17**	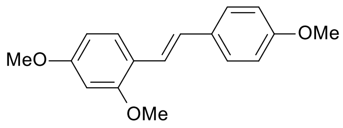	4′(OMe)-substituted
**18**	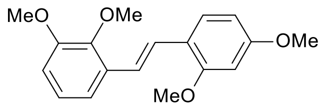	2′,3′-disubstituted
**19**	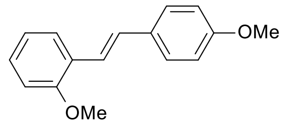	4′(OMe)-substituted
**20**	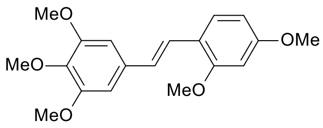	2,4-disubstituted
**21**	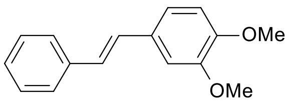	unsubstituted
**22**	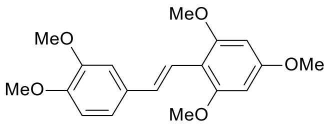	3,4-disubstituted
**23**	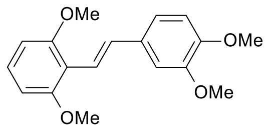	3,4-disubstituted
**24**	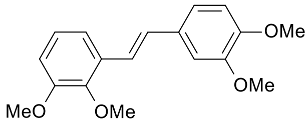	3,4-disubstituted
**25**	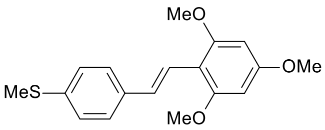	4(SMe)-substituted
**26**	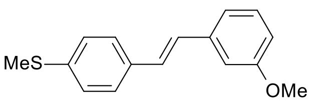	4(SMe)-substituted
**27**	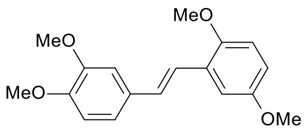	3,4-disubstituted

Compound	Structure	Ring close to HEME
28	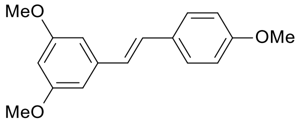	4′(OMe) –substituted
29	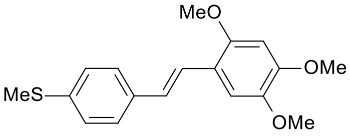	4(SMe)–substituted
30	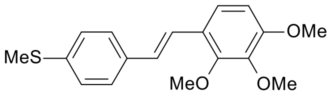	4(SMe)–substituted
31	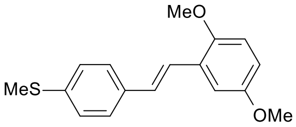	4(SMe)–substituted
32	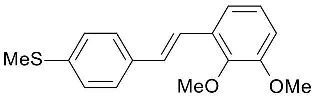	4(SMe)–substituted
33	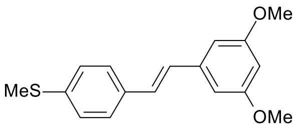	4(SMe)–substituted
34	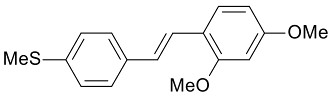	4(SMe)–substituted
35	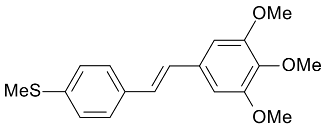	4(SMe)-substituted
36	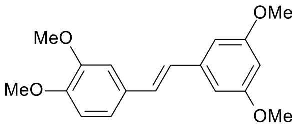	3,4-disubstituted
37	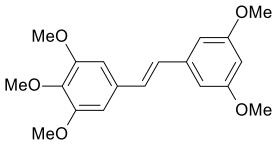	3′4′5′-trisubstituted
38	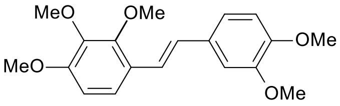	3,4-disubstituted

Compound	Structure	Ring close to HEME
**39**	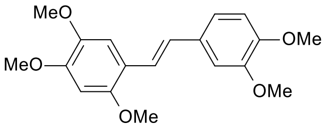	3,4-disubstituted
**40**	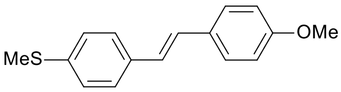	4(SMe)–substituted
**41**	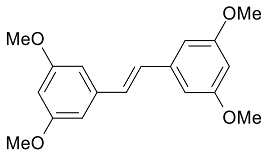	3,5-disubstituted
